# Increased incidence and improved survival in endometrial cancer in Sweden 1960–2014: a population-based registry survey

**DOI:** 10.1186/s12885-023-10746-0

**Published:** 2023-03-27

**Authors:** Filip Herbst, Paul W. Dickman, Louise Moberg, Thomas Högberg, Christer Borgfeldt

**Affiliations:** 1 Department of Obstetrics and Gynecology, Skåne University Hospital Lund, Lund University, Klinikgatan 12, 221 85 Lund, Sweden; 2 Department of Medical Epidemiology and Biostatistics, Karolinska Institute, Stockholm, Sweden; 3 Department of Cancer Epidemiology, Skåne University Hospital Lund, Lund University, Lund, Sweden

**Keywords:** Endometrial cancer, Endometrial carcinoma, Histopathology, FIGO stage, Long-term follow-up, Net survival, Population-based, Cancer registry, Incidence

## Abstract

**Background:**

An investigation of trends of incidence and net survival (NS) for endometrial cancer in Sweden.

**Methods:**

Morphologically verified endometrial carcinoma diagnosed 1960 to 2014 were collected from the nation-wide Swedish Cancer Registry. Endometrial cancer patients were assessed with regards to time trends for incidence and 54,825 cases remained for survival analyses. Cases diagnosed 1995 to 2014 were categorized according to detailed morphology and from 2005 to 2014 FIGO stage was also categorized.

**Results:**

There was a trend of increasing incidence of endometrial carcinoma for women above 55 years of age. NS was improved at 5- and 10-year follow-up. The 5-year net survival in 2010–2014 was 86%. The most prominent improvement in NS was found in the elderly women above 75 years of age.

**Conclusions:**

This study observed increased incidence of endometrial cancer in Sweden from 1960 to 2014. The progress in diagnostics and treatment, seem to have improved the net survival, especially in elderly women.

## Background

Uterine corpus carcinoma, or endometrial carcinoma, is the 6th most common cancer in women in Sweden, and worldwide. Globally the incidence is increasing mainly due to increased life expectancy and obesity [1]. In Sweden approximately 1400 women are diagnosed annually [2, 3]. Thanks to early symptoms and diagnosis, the survival rate is high. In Sweden, in 2016 the 5-year and 10-year survival rates were 84.2% and 80.7% respectively [3]. The Swedish Cancer Registry has been collecting national data since 1958. The registration of malignant, premalignant and certain benign tumors is mandatory and more than 96% of new cases are registered [4].

Endometrial carcinomas are often categorized into two groups, type I and type II. Type I, which is the most common (80–90%), has a better prognosis, is hormone dependent and is associated with hyperestrogenism. Type I has a high to intermediate differentiation (grade 1–2) and endometrioid morphology. By contrast, type II (10–20%) is generally hormone independent with worse prognosis. Type II includes endometrioid cancer with low degree of differentiation (grade 3), serous carcinoma, clear cell cancer, carcinosarcomas/ malignant mixed Müllerian tumor, dedifferentiated and undifferentiated cancer [2].

There are several risk factors associated with endometrioid endometrial cancer including obesity, diabetes, hypertension and nulliparity [1, 2]. Unopposed estrogen treatment increases the risk while estrogen treatment in combination with continuous progesterone, such as in combined contraceptives has been proven to be protective. [1, 2, 5, 6]. Tamoxifen treatment in breast cancer patients has also been shown to increase the risk of endometrial cancer [1, 2].

The most important prognostic factor in endometrial cancer is stage. Stage also determines recommended treatment. In Sweden staging is performed according to the International Federation of Gynecology and Obstetrics (FIGO) surgical staging system stage I-IV, with subcategories. However, registration of stage was not introduced into the Swedish Cancer Registry until 2004.

Surgery is the keystone in the treatment of endometrial cancer [1]. Adjuvant radiation therapy has not been proven effective on overall survival but has been shown to reduce pelvic recurrence [7, 8]. Adjuvant chemotherapy on the other hand has been found to increase survival and is recommended for patients where the risk of recurrence is significant. [9].

The aim of this study was to investigate long term trends of incidence and net survival (NS) for endometrial cancer in Sweden, and in later time periods with regards to stage and morphology.

## Methods

The population-based nationwide Swedish Cancer Registry started registration in 1958. The registry contains data about gender, personal identification number, domicile, date of diagnosis, tumor site, histological type, stage at diagnosis, basis of diagnosis, reporting hospital and department. The Swedish Cancer Registry receives data once yearly from six regional registries associated with regional cancer centers covering the whole country. It is compulsory for clinicians, pathologists and cytologists to independently register all patients with premalignant and malignant conditions and certain benign tumors. This secures the high coverage of the registry [10]. The tumor site was in 1958–1986 coded according to International Classification of Diseases—7 (ICD-7), 1987–1992 according to ICD-9, 1993–2004 according to International Classification of Disease of Oncology 2 (ICD-O/2) and from 2005 according to ICD-O/3. The codes were translated to ICD-7 for the entire time-period to enable comparability over time. Morphological codes are available during the whole period as the older World Health Organization histology code (WHO/HS/CAN/C24.1). A more detailed morphology coding according to ICD-O/2 was used 1993 to 2004, and since 2005 ICD-O/3 has been used. Since 2004 information about stage has been collected [10]. The completeness of the Swedish Cancer Registry is over 96% [4] and nearly all uterine cancer cases were verified by morphology [10]. Since Swedish residents all have a unique identification number, the follow-up of the patients in the registry is close to complete until time of death or emigration [11]. This was done by linking the data with data from the Swedish Cause of Death Registry using the Swedish personal identification numbers.

**Fig. 1:**
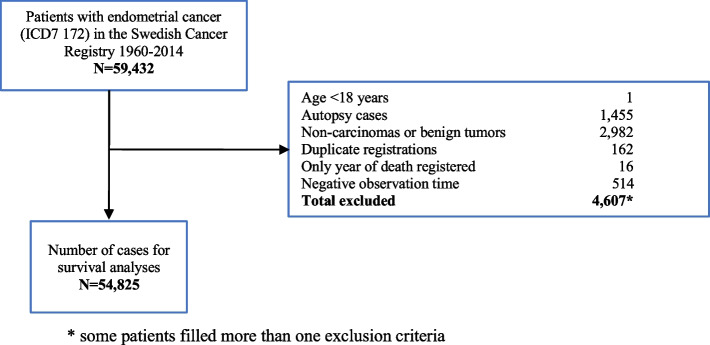
Flow chart of patients who met inclusion and exclusion criteria for survival analyses

Data from 59,432 cases of endometrial carcinomas (ICD-7 172) from 1960 to 2014 was retrieved from the Swedish Cancer Registry and assessed with regards to time trends for incidence. Sarcomas and carcinosarcomas are registered under site code ICD-7 174, why they were not included. Of the retrieved patients 4,607 cases were excluded based on the following exclusion criteria; age < 18 years old, autopsy cases, cases without PAD/cytology, benign morphology or non-carcinomas, duplicate registrations, only year of death registered, zero or negative observation time (Fig. 1). These exclusion criteria left 54,825 cases of endometrial carcinomas for survival analyses.

### Statistical analyses

Incidence rates were age-standardized to the World Standard population 2000–2025 to facilitate international comparisons [12]. Survival time was measured from date of diagnosis until date of death, date of emigration, or May 7^th^ 2020. We estimated net survival in a relative survival framework, which is standard approach for population-based studies of cancer patient survival [13]. Net survival is the probability of surviving beyond a given time without dying of the disease under study in a hypothetical scenario where the disease under study is the only possible cause of death. Net survival is preferred for comparing cancer patient survival between countries or within a single country since it is independent of mortality due to other causes. There are two frameworks available to estimate net survival; relative- and cause-specific. Relative survival is generally favored for population-based studies [14].

We estimated net survival using flexible parametric models [15]. Expected mortality rates for women, stratified by age and calendar year, were retrieved from the Human Mortality Database (http://www.mortality.org) based on data from Statistics Sweden. The model included the main effects of age at diagnosis (categorized as 18–44, 45–54, 55–64, 65–74, and > 75 years), morphology (high risk versus low risk; Table 1), year of diagnosis (1960–2014) as restricted cubic spline with 3 degrees of freedom, all three two-way interactions between age, year of diagnosis, and morphology, and time-varying effects of age, morphology, age*morphology and age*year. The baseline cumulative excess hazard was modelled as a restricted cubic spline with 5 degrees of freedom and the time-varying effects were modelled using 2 degrees of freedom. Based on this model, we estimated temporal trends in net survival within each age group along with age-standardized net survival using the International Cancer Survival Standard (ICSS) population number 1 [16]. We also estimated the difference in age-standardized net-survival between the two morphologic types, and relating to stage and morphologic types. The analytic process for age-standardization is illustrated (using publicly available data for melanoma where the exposure of interest is sex rather than morphology) at http://pauldickman.com/software/stata/age-standardise-standsurv/. The statistical analyses were performed using Stata version 17 (StataCorp, TX, USA). For comparisons of grouped data, the chi2-analysis was used.

**Table 1:** Morphology related to time periods

Morphology	ICD-O/2	1995–99	2000–04	2005–09	2010–14	Total
Adenocarcinoma (a)	8140/3	5,460 (92.0%)	5,972 (93.2%)	6,183 (92.4%)	6,001 (88.4%)	23,616 (91.5%)
Papillary serous cystadenocarcinoma (b)	8460/3	116 (2.0%)	150 (2.3%)	220 (3.3%)	332 (4.9%)	818 (3.2%)
Clear cell adenocarcinoma (b)	8310/3	84 (1.4%)	88 (1.4%)	102 (1.5%)	167 (2.5%)	441 (1.7%)
Serous cystadenocarcinoma (b)	8441/3	1 (0.02%)	3 (0.1%)	11 (0.2%)	168 (2.5%)	183 (0.7%)
Carcinoma, undifferentiated (b)	8020/3	37 (0.6%)	46 (0.7%)	40 (0.6%)	35 (0.5%)	158 (0.6%)
Adenosquamous carcinoma (a)	8560/3	57 (1.0%)	59 (0.9%)	30 (0.5%)	10 (0.2%)	156 (0.6%)
Papillary adenocarcinoma (a)	8260/3	80 (1.4%)	35 (0.6%)	18 (0.3%)	3 (0.04%)	136 (0.5%)
Mucinous adenocarcinoma (a)	8480/3	43 (0.7%)	14 (0.2%)	39 (0.6%)	20 (0.3%)	116 (0.5%)
Endometrioid adenocarcinoma (a)	8380/3	22 (0.4%)	14 (0.2%)	9 (0.1%)	19 (0.3%)	64 (0.3%)
Carcinoma (b)	8010/3	10 (0.2%)	10 (0.2%)	18 (0.3%)	17 (0.3%)	55 (0.2%)
Squamous cell carcinoma (b)	8070/3	6 (0.1%)	10 (0.16%)	9 (0.1%)	4 (0.1%)	29 (0.1%)
Adenocarcinoma with squamous metaplasia (a)	8570/3	13 (0.2%)	2 (0.03%)	7 (0.1%)	1 (0.01%)	23 (0.1%)
Neuroendocrine carcinoma (b)	8246/3	0 (0.0%)	0 (0.0%)	1 (0.01%)	7 (0.1%)	8 (0.03%)
Mucinous cystadenocarcinoma (a)	8470/3	1 (0.02%)	1 (0.02%)	0 (0.0%)	5 (0.1%)	7 (0.03%)
Small cell carcinoma (b)	8041/3	0 (0.0%)	1 (0.02%)	3 (0.04%)	1 (0.01%)	5 (0.02%)
Carcinoma, anaplastic (b)	8021/3	1 (0.02%)	0 (0.0%)	1 (0.01%)	1 (0.01%)	3 (0.01%)
Mesonephroma, malignant (c)	9110/3	2 (0.03%)	0 (0.0%)	0 (0.0%)	0 (0.0%)	2 (0.01%)
Adenoid cystic carcinoma (c)	8200/3	0 (0.0%)	1 (0.02%)	0 (0.0%)	0 (0.0%)	1 (0.0%)
Cystadenocarcinoma (a)	8440/3	0 (0.0%)	0 (0.0%)	1 (0.01%)	0 (0.0%)	1 (0.0%)
Large cell carcinoma (c)	8012/3	0 (0.0%)	0 (0.0%)	1 (0.01%)	0 (0.0%)	1 (0.0%)
Transitional cell carcinoma (c)	8120/3	0 (0.0%)	0 (0.0%)	1 (0.01%)	0 (0.0%)	1 (0.0%)
Total		5,933 (100.0%)	6,406 (100.0%)	6,694 (100.0%)	6,791 (100.0%)	25,824 (100.0%)

(a) Low risk (*n* = 24,119), (b) High Risk (*n* = 1,700), (c) Other (*n* = 5)

## Results

In total 59,432 cases of endometrial carcinomas, diagnosed from 1960–2014, meeting the inclusion criteria, were identified. After exclusions (Fig. 1) 54,825 remained for survival analyses.

**Fig. 2:**
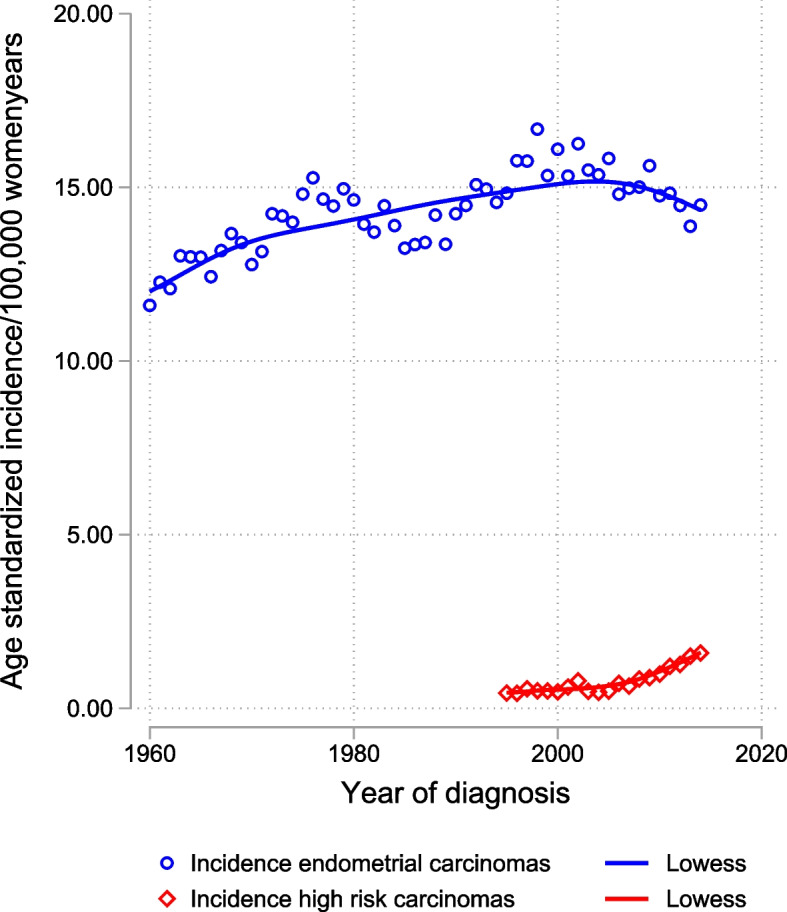
Age-standardized incidence of endometrial carcinomas per 100,000 women standardized to the world population. High risk morphology (as specified in Table 1 (b)) also shown separately from 1995–2014. Lowess = locally weighted scatterplot smoothing

**Fig. 3:**
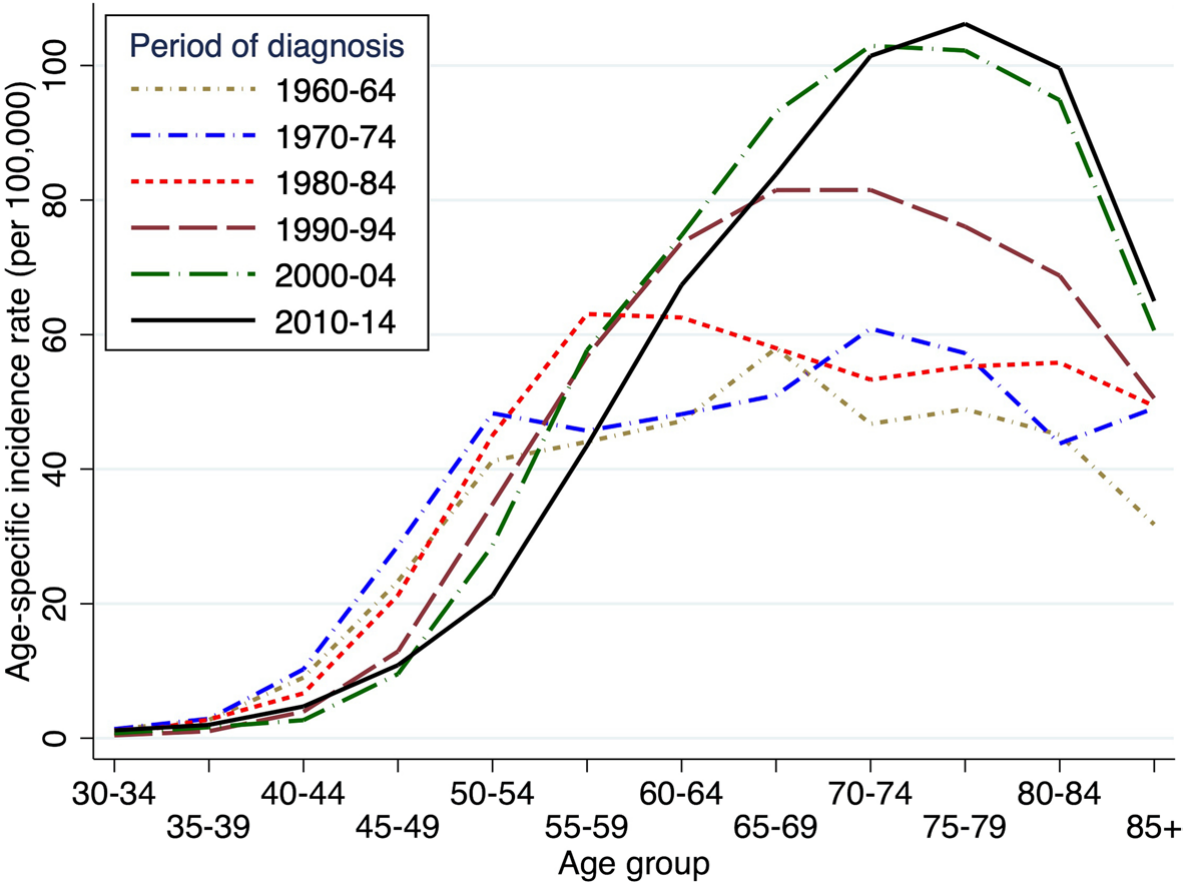
Age-specific incidence rates of endometrial cancer per 100,000 women according to time periods

The number of endometrial cancer cases doubled during the studied time period (Table 2) and the crude incidence increased from 16.2/100 000 women in 1960 to 28.6/100 000 in 2014. The share of cases in the younger age groups, < 55 years of age, have decreased over time from 31% in the first decade, to 9% at the end of the study period. The decrease was also found in absolute numbers despite an increasing population. The increase in incidence was also observed after age standardization to the World Health Organization 2000–2025 standard population, from 1960 to 2005, after which there was a slight decrease (Fig. 2). High risk endometrial cancers such as clear cell adenocarcinomas and serous carcinomas increased in incidence since 2005 (Fig. 2). There was a gradual shift in age at onset (Fig. 3). The age-specific incidence decreases in the younger age groups but increases substantially in women above 60 years of age.

**Table 2:** Number of women diagnosed with endometrial carcinoma, and eligible for survival analyses in age-groups related to time periods

	**1960-69**	**1970-79**	**1980-89**	**1990-99**	**2000-09**	**2010-14**	**TOTAL**
**MEDIAN AGE (RANGE)**	60 (23–95)	62 (22–97)	64 (24–96)	68 (20–105)	69 (23–104)	69 (25–99)	66 (20–105)
**AGE GROUPS:**							
**18–44**	337 (5.0%)	312 (3.8%)	250 (2.8%)	168 (1.5%)	178 (1.4%)	120 (1.8%)	1,365 (2.5%)
**45–54**	1,772 (26.3%)	1,946 (23.8%)	1,327 (15.0%)	1,315 (11.8%)	1,057 (8.1%)	487 (7.2%)	7,904 (14.4%)
**55–64**	2,107 (31.3%)	2,557 (31.3%)	2,875 (32.5%)	2,916 (26.1%)	3,478 (26.6%)	1,613 (23.8%)	15,546 (28.4%)
**65–74**	1,698 (25.2%)	2,130 (26.0%)	2,611 (29.5%)	3,690 (33.0%)	4,087 (31.2%)	2,348 (34.6%)	16,564 (30.2%)
**75 +**	816 (12.1%)	1,234 (15.1%)	1,783 (20.2%)	3,090 (27.6%)	4,300 (32.8%)	2,223 (32.7%)	13,446 (24.5%)
**TOTAL**	6,730 (100.0%)	8,179 (100.0%)	8,846 (100.0%)	11,179 (100.0%)	13,100 (100.0%)	6,791 (100.0%)	54,825 (100.0%)

In 2010–2014 80% of the patients were diagnosed in stage I or II (Table 3). There was an improvement in registration of stage between the two time periods with the percentage of patients with missing stage decreasing from 21 to 5%. There was a decrease in stage II (*p* < 0.0001) and a slight increase in stage I cases (*p* < 0.0001). There was also an increase in stage IV cases (*p* < 0.0001).

**Table 3:** FIGO stage of endometrial carcinoma related to time periods. Difference between the two time periods is shown with non stage cases (missing) excluded to enable comparisons

**FIGO STAGE**	**2005-09**	**2010-14**	**TOTAL**	DIFFERENCE*
**I**	3859 (57.7%)	4924 (72.5%)	8783 (65.1%)	+ 3.2% (*p* < 0.0001)
**II**	721 (10.8%)	517 (7.6%)	1238 (9.2%)	- 5.6% (*p* < 0.0001)
**III**	507 (7.6%)	640 (9.4%)	1147 (8,5%)	+ 0.3% (*p* = 0.56)
**IV**	203 (3.0%)	382 (5.6%)	585 (4.3%)	+ 3.1% (*p* < 0.0001)
**MISSING**	1404 (21.0%)	328 (4.8%)	1732 (12.9%)	
**TOTAL**	6694 (100.0%)	6791 (100.0%)	13,485 (100.0%)	

^*^Difference between the two periods in percentage in each stage (excluding missing) and p-value for test of statistical significance within each stage

**Fig. 4:**
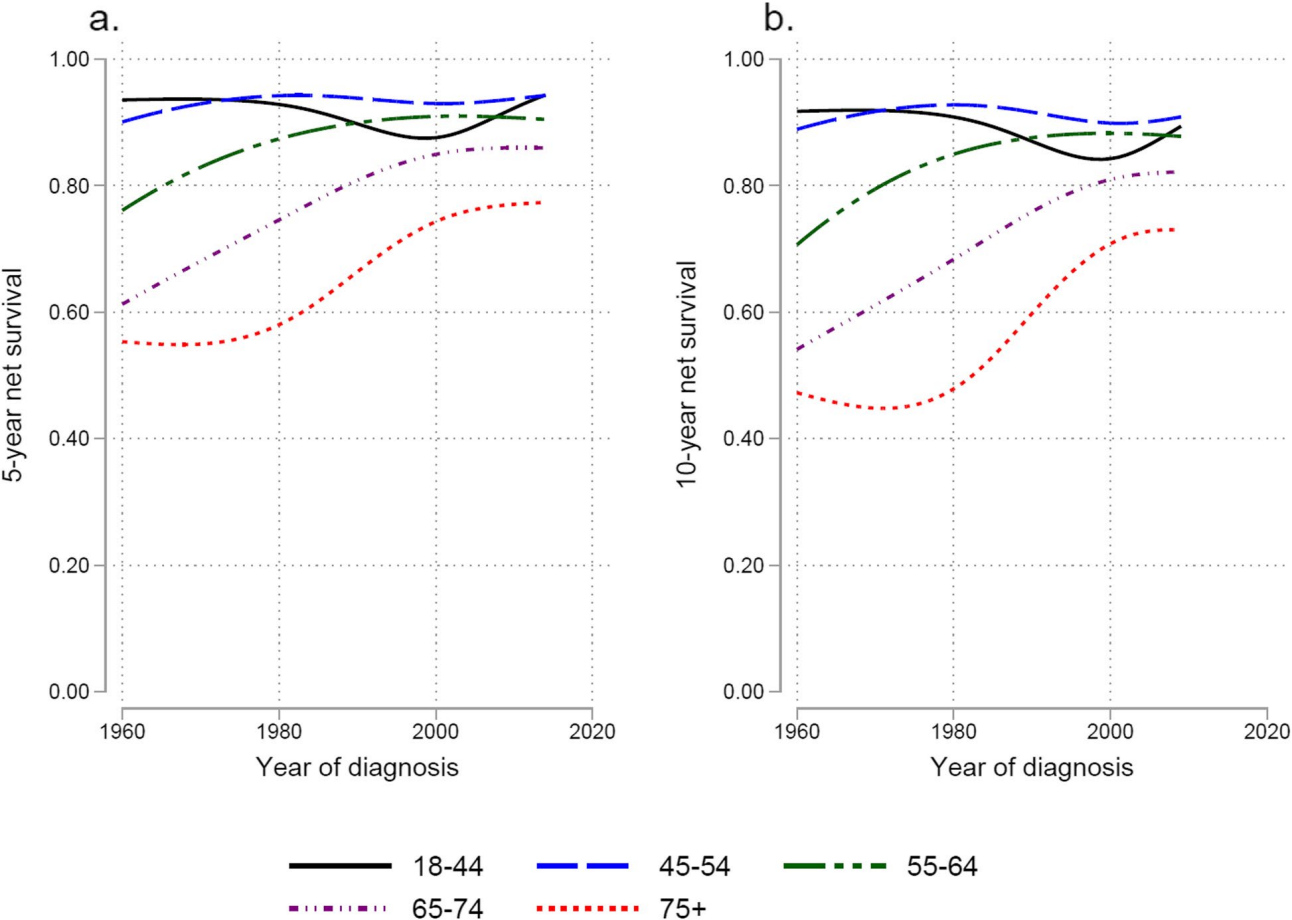
Time trends for 5- (**a**.) and 10-year (**b**.) net survival according to age groups

There was a trend of improved survival over the entire study period regarding both 1-, 2- (not shown), 5- and 10-year net survival (NS) rates for women above 55 years of age (Fig. 4). The youngest age groups have excellent survival during the whole period. For women under 45 years of age there was a drop in NS during the 1990s.

**Fig. 5:**
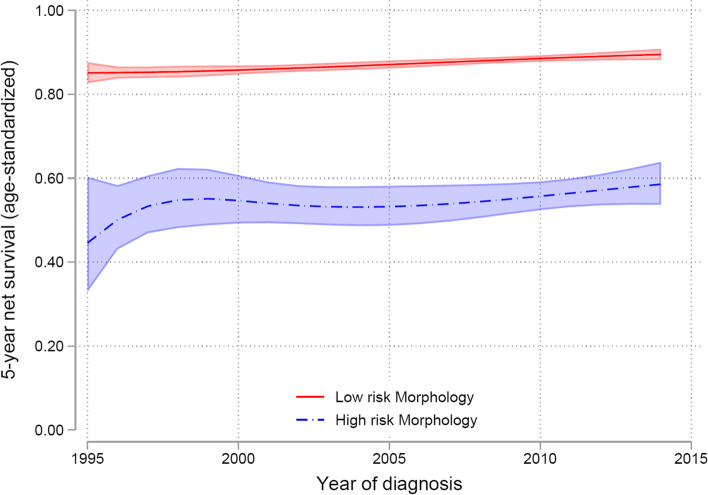
Trends for 5-year net survival standardized to the International Cancer Survival Standard (ICCS-1) for low risk versus high-risk morphology. 95% confidence intervals are shown as shadowed areas

The survival trends for low- and high risk morphologies are shown in Fig. 5. There is a great difference in 5-year NS for low- and high risk morphologies with low risk morphologies approaching 90%, and high risk morphologies approaching 60% 5 year NS at the end of the period.

**Fig. 6:**
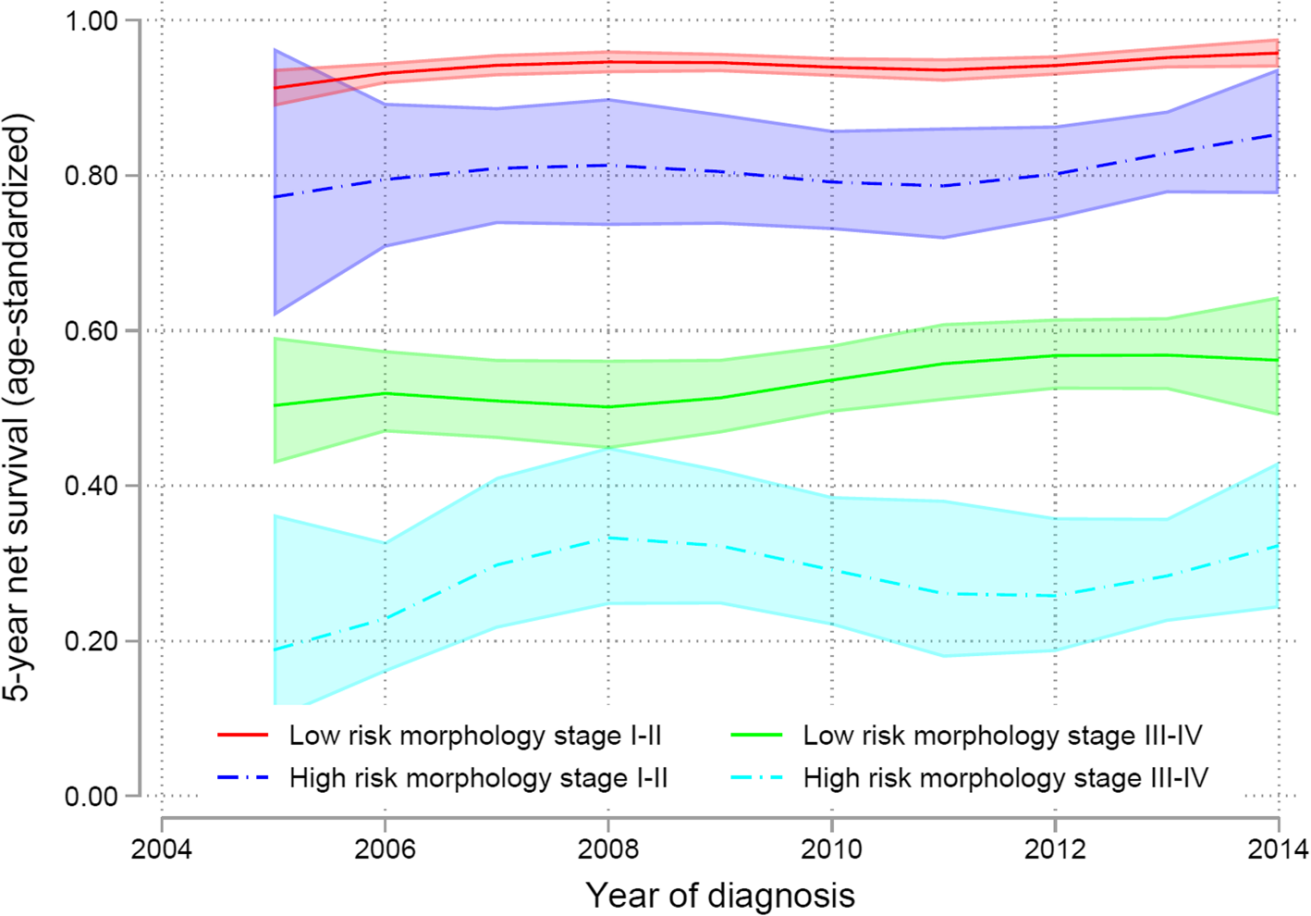
Trends for 5-year net survival standardized to the International Cancer Survival Standard (ICCS-1), for low risk versus high-risk morphology, and early FIGO stage I-II and advanced FIGO stage III-IV. 95% confidence intervals showed as shadowed areas

The NS is significantly worse for high risk morphology patients also when separated into stage I-II versus stage III-IV (Fig. 6). 5-year NS in with low risk morphology stage I-II is approximately 95%, and in high risk morphology stage III-IV only 30%.

**Fig. 7:**
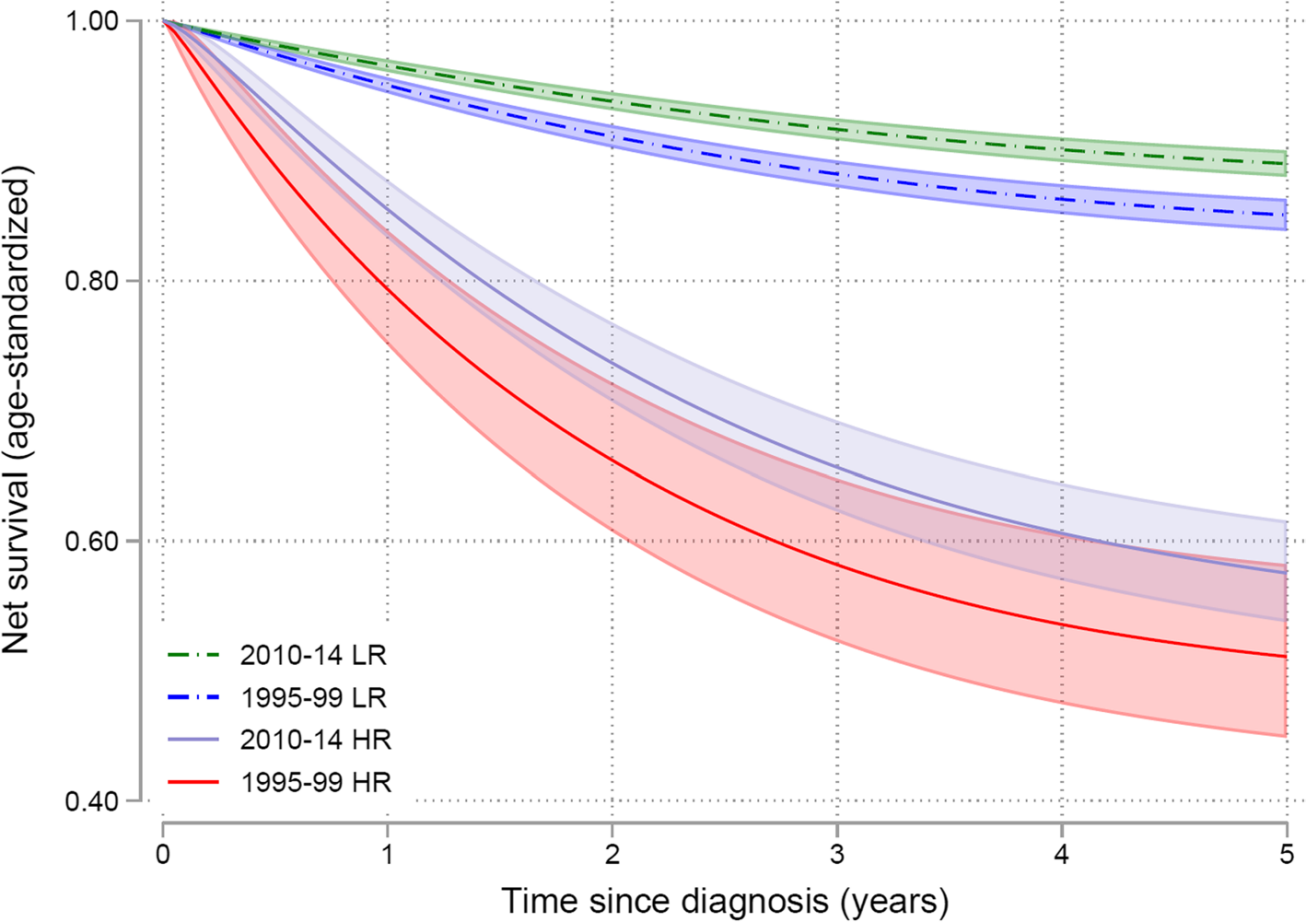
Net Survival (lines) with confidence intervals (shadowed areas) in low- risk morphology (endometrioid endometrial cancer) compared with high-risk morphology (serous and clear cell endometrial cancer)

The majority of excess mortality occurred during the first three years after diagnosis for both low risk morphology patients (endometrioid endometrial cancer) and high-risk morphology patients (non-endometrioid endometrial cancer) (Fig. 7). The NS in endometrioid patients was improved from 1995–1999 to 2010–2014 with NS of approximately 89% at 5 years and only slightly decreased during further follow up. In morphological high-risk tumors, the NS was not significantly increased, approximately 57% at 5 years.

## Discussion

This study observed an increased incidence of endometrial cancer from 1960 to 2005, and thereafter a small decline. Increased incidence has also been shown in other European countries such as the UK and Norway [17, 18]. This is however not consistent with for example the US, where incidence declined 1975–1987, was stable 1987–2006 and increased slightly 2007–2017[19]. In Denmark incidence increased 1943–1983 after which it decreased somewhat, and thereafter stabilized [18].

The net survival improved gradually from the 1960s to 2014 with a 5 year NS increasing from 69% to 86%. The most prominent improvement in NS was found in the elderly women above 75 years of age. Improved survival has also been seen in Norway, Denmark and Finland [18]. In contrast there has been a decrease in survival in the United States [20].

There are several factors that likely contribute to the increase in incidence of endometrial cancer during the study period. The average lifespan in women increased from 74.9 to 84.1 years and the population above 65 years increased by a factor of 2.3 [21]. The median age at diagnosis increased from 60 to 69 years of age. The number of cases of endometrial cancer doubled during the studied period while the female population during this time increased only by 30% [21]. The ageing population affects the increase in crude incidence, but the increase is also observed in the age standardized incidence. There is a general shift over time towards diagnosis at an older age, with not only an increasing incidence at older ages but also a decrease of cases in women less than 55 years of age, where the decrease is not only in shares but also in absolute numbers. This is not likely to be explained by the use of combined oral contraceptives considering their long-term protective potential [5]. The protective effect should then likely be seen also at older ages. There is however a trend towards use of oral contraceptives and intrauterine devices with progestins at younger ages which may reduce the incidence [22]. Age standardization to World Health Organization 2000–2025 standard population was chosen to facilitate international comparisons. Since the Swedish population is significantly older, the incidence would have been greater if we had chosen to age standardize to a Swedish population. Furthermore, the upward shift in age at diagnosis (shown in Fig. 3) affects the increase in incidence. Since incidence is decreased at lower ages and increased at higher ages the increase in incidence is not as visible when the material is age standardized to a younger population such as the World Health Organization 2000–2025 standard population.

There have been significant changes in the prevalence of risk factors over time. Obesity increases among Swedish women and the prevalence of body mass index of ≥ 30 kg/m^2^ has increased from 7% in 1985 to 11–13% in 2000 and 17% in 2012 [23, 24]. Parity has decreased from an average of 2,17 children/woman in 1960 to 1,88 in 2014 [21]. Smoking has been shown to be protective against endometrial cancer [1, 2] and the rate of smoking has gradually decreased in Sweden during the study period [25]. Sales of menopausal hormone therapy increased rapidly in Sweden from the late 1980s to peak in 1999 and thereafter declined dramatically until 2017 [26]. Of menopausal hormone therapy sold in 1999, 59% was as fixed combinations of estrogen and gestagens and 41% as unopposed estrogen or in combination with gestagen via separate prescriptions [26]. The changes in menopausal hormone therapy cannot clearly be seen to affect the incidence in this material. This may be due to the fact that unopposed estrogen was not widely spread, or the effect may be hidden in other changes over time.

Improved diagnostical efficacy with transvaginal ultrasonography and endometrial biopsies have probably contributed to the increased incidence with fewer missed endometrial cancer cases. The registered increase in clear cell carcinomas and serous carcinomas is likely a reflection of a shift in morphological diagnostics. Diagnostic advances have been made especially in immunohistochemistry. P53 helps to identify serous adenocarcinoma [27] and Napsin A may be used as a marker for clear cell carcinomas [28].

There has been variation over time in the number of hysterectomies performed on benign indications, which affects the incidence of endometrial cancer, since a previous hysterectomy protects from the disease. There was an increase in hysterectomies performed from 178/100 000 in 1987 to 232/100 000 in 1999. After this, the number of hysterectomies went down to 210/100 000 in 2003 and has thereafter continued to decrease by another 16% until 2019 [29, 30]. The peak in hysterectomies was around the same time there was a peak in prescribed menopausal hormone therapy.

When comparing the distribution of stage over time there was a decrease in stage II and an increase in stage I cases (Table 3). There was also an increase in stage IV cases. These increases are still obvious when comparing shares of the staged cases, excluding the unstaged cases to eliminate the bias of a larger staged share in the later period. The FIGO stage I cases then increased from 72.95% to 76.19% and FIGO stage IV cases from 3.84% to 5.91%. The lack of completeness in reporting stage during 2005–2009 compared with 2010–2014 makes conclusions more uncertain, but the decrease in stage II cases can logically not be a result of underreporting during the first period. The decrease in stage II- and increase in stage I-cases may be due to earlier diagnosis before progression from stage I. If this is true, coming studies should evaluate reasons for patients and doctors delay in patients with stage II or more to improve early discovery. The increase in stage IV could be due to improved diagnostics causing a shift upwards in stage when signs of spread become visible with improved imaging, such as more frequent use of CT-scan.

The trend of increased NS was observed for the whole study period. For reasons unknown, but maybe due to patients’ and doctors’ delay, the improved NS could not be found in the youngest patients. Since this group is small, the overall NS has however greatly improved. There have been several advances in the treatment of endometrial cancer during the study period, such as introduction of platinum-based chemotherapy in the 1980s [31]. The long-term continuing improvement is likely a combination of many small improvements happening over time, ranging from improved knowledge of prognostic factors and their importance in choice of treatment, to improved surgical methods gradually being implemented [32]. In Sweden the treatment of endometrial cancer has traditionally been characterized by a liberal use of both preoperative and postoperative radiation [33]. This tradition was broken by a treatment program in the Southern Health Care Region 1993 to 1996 [34], which minimized radiation therapy and showed no detrimental effect on survival. Since then, the use of adjuvant radiotherapy has gradually diminished and is reserved to a rather small fraction of patients with adverse prognostic factors. Other changes in treatment came after the implementation of the first Swedish National Guidelines for endometrial cancer in 2011. Preoperative high-risk tumors, defined as non-endometrioid histology (serous, clear cell carcinoma or carcinosarcoma), endometrioid adenocarcinoma FIGO grade 3, or non-diploid tumors, were recommended to be treated with a lymphadenectomy of the pelvic and para-aortic regions (up to the left renal vein) in addition to hysterectomy and salpingo-oophorectomy. Postoperatively, patients with high-risk histology and negative nodes were recommended chemotherapy ± brachytherapy and those with positive nodes or no lymphadenectomy were offered chemotherapy + external beam radiotherapy.

There were substantial differences in NS for different age groups with worse prognosis in the elderly, even though other causes of death were excluded in this way of analyzing NS. Similar observations have been made for other cancers, and a performance status with co-morbidity in older age may contribute to patients not being able to cope with treatment or complications of treatment as well as younger individuals [35]. It has previously been shown that the median age at onset is higher in type II endometrial cancer, which has a higher mortality rate [36], which likely contributes. Women above 75 years of age showed the greatest improvement of NS over time which may be accounted to improved treatment of co-morbidity and refined treatment both with minimal invasive surgery as well as improved chemotherapy treatment [34]. Improved anesthesiological methods and a changed attitude towards treatment of old women may also have contributed to improved NS in the older women. The lesser survival rates for women under 45 years of age might be due to delayed diagnosis, since abnormal bleedings before menopause may be less likely to be investigated.

This study is a nationwide, population-based study spanning 55 years, which combined with the high quality of the Swedish Cancer Register, with > 96% coverage and very high morphological verification in the later period of the study [4, 12], are major strengths. Detailed morphology was not registered before 1993 and stage not before 2004. Therefore, trends for specific morphologies and stages could not be assessed for the full study period. Furthermore, the absence of central pathology is a limitation of the study.

There have been various changes in the classification of especially uterine sarcomas, why sarcomas were excluded. Carcinosarcomas are nowadays regarded and classified as high-risk carcinomas [1, 37]. They are however registered in the Cancer Registry together with sarcomas under site code ICD-7 174. Carcinosarcomas make up less than 5% of all uterine malignancies in other studies [37] and in the Swedish Quality Register for Gynecological Cancer 2010–2018 carcinosarcomas accounted for 3% of the endometrial cancers (personal communication).

## Conclusions

The incidence of endometrial cancer in Sweden has increased over the last 60 years. However, with improvement in treatment of the disease, and possibly due to earlier diagnosis, net survival has improved substantially among women aged 55 years and older at diagnosis.

## Data Availability

The data that support the findings of this study are available from the Swedish Cancer Registry, but restrictions apply to the availability of these data, which were used under license for the current study, and so are not publicly available.

## Abbreviations

FIGO: Fédération Internationale de Gynécologie et d'Obstétrique / International Federation of Gynecology and Obstetrics
ICD: International classification of diseases
NS: Net survival
